# The Importance of l-Arginine:NO:cGMP Pathway in Tolerance to Flunitrazepam in Mice

**DOI:** 10.1007/s12640-016-9688-3

**Published:** 2016-12-12

**Authors:** Sylwia Talarek, Joanna Listos, Jolanta Orzelska-Gorka, Malgorzata Jakobczuk, Jolanta Kotlinska, Grazyna Biala

**Affiliations:** Chair and Department of Pharmacology and Pharmacodynamics, Medical University of Lublin, Chodzki 4A, 20-093 Lublin, Poland

**Keywords:** Nitric oxide, Flunitrazepam, Motor impairment, Tolerance, mice

## Abstract

The goal of the study was to investigate the effects of drugs modifying l-arginine:NO:cGMP pathway on the development of tolerance to flunitrazepam (FNZ)-induced motor impairment in mice. FNZ-induced motor incoordination was assessed on the 1st and 8th days of experiment, using the rotarod and chimney tests. It was found that (a) both a non-selective nitric oxide synthase (NOS) inhibitor: *N* ^G^-nitro-l-arginine methyl ester (l-NAME) and an unselective neuronal NOS inhibitor: 7-nitroindazole (7-NI) inhibited the development of tolerance to the motor-impairing effects of FNZ in the rotarod and the chimney tests and (b) both a NO precursor: l-arginine and a selective inhibitor of phosphodiesterase 5 (PDE5): sildenafil did not affect the development of tolerance to FNZ-induced motor impairment in mice. Those findings provided behavioural evidence that NO could contribute an important role in the development of tolerance to FNZ in mice.

## Introduction

Benzodiazepines (BZ) are compounds with sedative, anxiolytic, anticonvulsant and muscle relaxant properties in humans and animals which were introduced to clinical practice over 50 years ago. This group of agents is widely used in clinics, but the use of these compounds is limited by the development of tolerance to most of their pharmacological actions that occurs after prolonged administration (Bateson 2002; Ferreri et al. 2015; Gravielle 2016).

Tolerance is defined as the reduction in certain pharmacological effects of a drug on repeated exposure to a given dose or the need to increase the amount of drug intake to obtain the same effect. In the case of BZ, tolerance develops with long-term use, even at low to moderate (i.e. therapeutic) doses and is characterized to many pharmacological effects of these drugs (including their sedative, anxiolytic, muscle relaxant and anticonvulsant effects) (Allison and Pratt 2003). All BZ have the capacity to promote the binding of the major inhibitory neurotransmitter γ-aminobutyric acid (GABA) to the GABA_A_ receptors (Smith et al. 2012). However, the neurobiological mechanisms underlying tolerance to BZ have not been well characterized yet.

Nitric oxide (NO), an important bioregulatory molecule, is synthesized from l-arginine, by a reaction catalyzed by nitric oxide synthase (NOS) (Bruckdorfer 2005). There are four members of the NOS family: neuronal (nNOS), endothelial (eNOS), inducible (iNOS) and mitochondrial NOS (mtNOS). nNOS and eNOS are Ca^2+^-calmodulin-dependent and are constitutively expressed in mammalian cells. NO allosterically interacts with soluble guanylyl cyclase (sGC) to increase cyclic guanosine 3′,5′-monophosphate (cGMP) expression and cGMP-dependent signaling. cGMP, in turn, modulates the activity of cGMP-dependent kinases, cGMP-gated ion channels and cGMP-regulated phosphodiesterases (PDE) (Bruckdorfer 2005). NO is supposed to play an important role in several brain functions and/or dysfunctions, including regulation of neuronal excitability, synaptic plasticity, anxiety, seizure activity and drug tolerance.

There are data pointing to the relationship between l-arginine:NO:cGMP pathway and GABA-mediated transmission in the central nervous system (CNS). For example, histochemical mapping of NOS revealed co-localization of NOS-positive neurons with GABA in the cerebral cortex and spinal cord (Valtschanoff et al. 1992). It has also been reported that NO is released as a result of activation of GABAergic neurotransmission in animal cortex (Guevara-Guzman et al. 1994; Segovia et al. 1994; Mantelas et al. 2003). It is also known that NO regulates the GABA release and uptake in the CNS (Guevara-Guzman et al. 1994). Moreover, there is behavioural data indicating that inhibition of NOS prolongs the sleeping time induced by BZ (Talarek and Fidecka 2004) and enhances the anticonvulsant (Talarek and Fidecka 2003), antinociceptive (Talarek and Fidecka 2002) and anxiolytic (Quock and Nguyen 1992) effects of BZ. In addition, there are findings suggesting some role of l-arginine:NO:cGMP pathway in the development of diazepam-induced tolerance to its motor-impairing effect in mice (Talarek et al. 2008).

The present experiments were undertaken to investigate the effect of compounds which modulate the l-arginine:NO:cGMP pathway on the development of tolerance to flunitrazepam (FNZ)-induced motor impairment in mice. This was done by measuring locomotor coordination in FNZ-administered mice after chronic pretreatment with *N* ^G^-nitro-l-arginine methyl ester (l-NAME)—a nonselective inhibitor of the NOS isoforms, 7-nitroindazole (7-NI)—a preferential inhibitor of nNOS, l-arginine—a substrate for NO formation and sildenafil—a phosphodiesterase type 5 (PDE5) inhibitor that enhances the effects of NO by inhibiting cGMP degradation and is commonly used in erectile dysfunction. The ataxic effect of FNZ after its repeated administration was examined in the rotarod and chimney tests. The rotarod test is used for assessing not only motor coordination of rodents but also its sense of balance. The chimney test is generally used as complementary to other tests which determine muscle relaxant activity (Vogel 2008).

FNZ, a full BZ agonist, is a fast-acting hypnotic drug and despite its addictive properties is still prescribed in some countries to patients with insomnia. FNZ has also become popular among alcohol and drug abusers (Druid et al. 2001). Moreover, FNZ has become known as a date-rape drug because sexual predators use it to chemically incapacitate their victims (Druid et al. 2001). Clinical data indicate that FNZ differs from other BZ e.g. in the ability to induce severe aggressive and disinhibited behaviours (Daderman et al. 2002). Our previous behavioural investigations on interactions of NO and BZ showed no differences between diazepam (DZ) and chlordiazepoxide or clonazepam (Talarek and Fidecka 2003; Talarek and Fidecka 2004) but supported atypical properties of FNZ, especially in its influence on cognition (Orzelska et al. 2013; Orzelska et al. 2015; Orzelska-Gorka et al. 2016) and locomotor activity in rodents (Talarek et al. 2013). Considering these intriguing data, the choice of FNZ in our present studies seems to be justified.

## Materials and Methods

### Animals

We used male albino Swiss mice that had an initial weight of 20–25 g. Animals were housed in groups of 10 and maintained in a 12-h light-dark cycle at a controlled temperature (21 ± 1 °C). They received standard food (LSM, Poland) and tap water ad libitum*.* All experiments were conducted according to the National Institute of Health Guidelines for the Care and Use of Laboratory Animals (8th edition) and to the European Community Council Directive for the Care and Use of Laboratory Animals of 22 September 2010 (2010/63/*EU*) and were approved by the local ethics committee.

### Behavioural Testing Procedures

#### Motor Coordination Was Assessed Using Rotarod and Chimney Tests

The rotarod consists of a circular rod (2 cm in diameter) turning at a constant speed (18 rpm). Animals place on the rod naturally try to remain on the rod rather than fall onto the platform some 30 cm below. Before drug testing, the mice were trained daily for a 3-day period. For each training session, the mice were placed on a rotating rod for 3 min with an unlimited number of trials. Drug testing was conducted at least 24 h after the final training trial. The length of time the animal remained on the rod was recorded (a 60-s maximal trial was used for the test).

The chimney test is a simple test for tranquilizing and muscle relaxant activity and can be used as an additional test with other tests determining motor coordination. The animals had to climb backwards up a plastic tube (3 cm in inner diameter, 25-cm long). The mice were trained once daily for 3 days. Motor impairment was assessed as the inability of mice to climb backwards up the tube within 60 s. The length of time that the mice spent in the chimney was recorded (Vogel 2008).

#### Spontaneous Locomotor Activity

In order to avoid the risk of obtaining the false effects in the rotarod and chimney tests caused by a possible influence of the l-arginine:NO:cGMP pathway modulators on the locomotor activity, mice spontaneous locomotor activity was measured using round actimeter cages (Multiserv, Lublin, Poland; 32 cm in diameter, two light beams). Two photocell beams, located across the axis, automatically measured animal’s movements. Each mouse was placed individually into the cage for 5 min (Vogel 2008). Spontaneous locomotor activity was evaluated on the 1st and 7th days of the experiment 35 min after NO modulator administration which corresponds with the time interval analysed in the rotarod and chimney tests.

### Drugs and Injections

l-NAME, l-arginine (Sigma Chemicals, St. Louis, USA) and sildenafil citrate (Sigma Chemicals, St. Louis, USA) were dissolved in 0.9% saline solution. 7-NI (RBJ, Natick, USA) and FNZ (Sigma Chemicals, St. Louis, USA) were dissolved in 0.5% Tween-80 (1–2 drops), gently warmed and diluted with saline solution. Control animals were injected with corresponding vehicle. All drug suspensions/solutions were prepared immediately prior to use. l-NAME, 7-NI, l-arginine and sildenafil were administered intraperitoneally (ip). FNZ was injected subcutaneously (sc). The doses of drugs modifying l-arginine:NO:cGMP pathway were tested in our previous experiments (Talarek et al. 2008; Talarek et al. 2010), and those which did not affect the motor performance in mice were used in the present studies. All the substances were administered in a volume of 10 ml/kg body weight.

### Experimental Procedures

The development of tolerance to FNZ-induced motor impairment was induced according to our own method (Talarek et al. 2013). Mice were administered with FNZ (1 mg/kg) for eight consecutive days. The development of tolerance was determined by comparing the motor coordination of mice treated with 1 mg/kg FNZ for 8 days, tested 30 min after the last dose of FNZ, and mice treated with FNZ (1 mg/kg) on the first day of experiment. l-NAME (50 or 100 mg/kg), 7-NI (10 or 20 mg/kg), l-arginine (125 or 250 mg/kg) and sildenafil (5 or 10 mg/kg) were administered 5 min before the injection of FNZ (1 mg/kg) for 7 days of the experiment. On the 8th day of the experiment, all groups of mice received only 1 mg/kg of FNZ. Motor coordination was measured on the 1st and 8th days of the experiment using the rotarod and chimney tests.

### Statistics

Data were analysed by two-way analysis of variance (ANOVA) to test the effects of the two factors: the treatment (saline, FNZ and FNZ with l-arginine:NO:cGMP modulators) and the time (days 1 and 8) on the motor coordination. Post hoc comparisons were performed using the Bonferroni test. A *p* value less than 0.05 was considered statistically significant. Data are presented as the mean ± standard errors (SEM). Each group included eight mice.

## Results

### Influence of Chronic l-NAME Treatment on the Development of Tolerance to FNZ-Induced Motor Disturbances

**Fig. 1:**
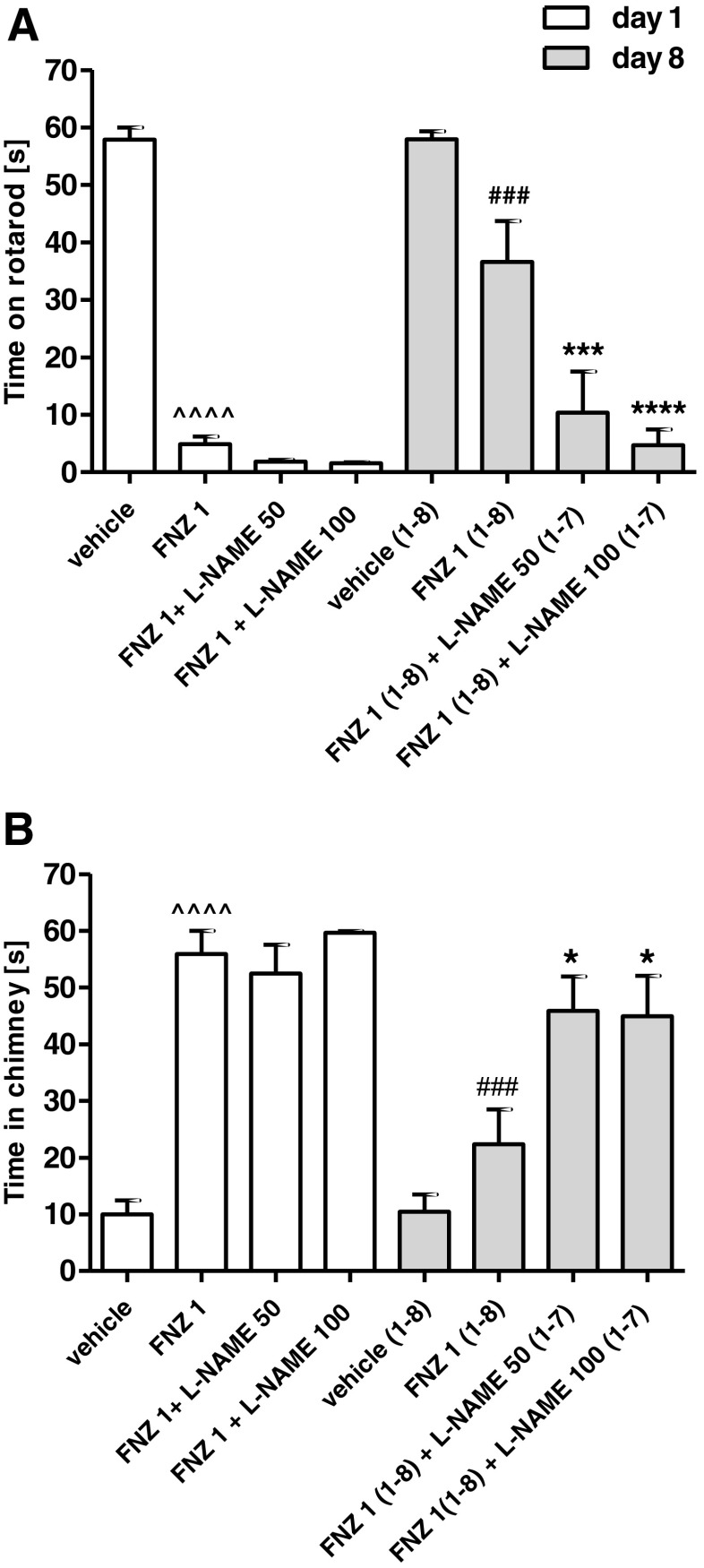
Influence of l-NAME treatment (50 or 100 mg/kg, days 1–7, ip) on the development of tolerance to FNZ-induced motor impairment (1 mg/kg, days 1–8, sc) as assessed by the rotarod test (**a**) and chimney test (**b**). Data represent the mean ± SEM of a group of eight mice (^^^^*p* < 0.0001 vs vehicle; ^###^ *p* < 0.001 vs FNZ 1; ****p < 0.0001; ***p < 0.01; *p < 0.05 vs FNZ 1 (1–8))

The two-way ANOVA indicated that a repeated administration of FNZ (1 mg/kg/day, 8 days) induced a significant treatment effect: *F*(3,56) = 86.26, *p* < 0.0001, time effect: *F*(3,56) = 16.09, *p* = 0.0002 and treatment × time interaction: *F*(3,56) = 7.01, *p* = 0004 in the rotarod test (Fig. 1a) and treatment effect: *F*(3,56) = 32.23, *p* < 0.0001, time effect: *F*(3,56) = 16.25, *p* = 0.0002 and treatment × time interaction: *F*(3,56) = 4.74, *p* = 0051 in the chimney test (Fig. 1b). The Bonferroni post hoc analysis revealed that the chronic administration of FNZ significantly reduced motor disturbances induced by an acute dose of FNZ, both in the rotarod (*p* < 0.0001) and the chimney tests (*p* < 0.0001). Those results indicate the development of tolerance to motor impairment after 8-day treatment of FNZ.

As shown in Fig. 1, the Bonferroni post hoc test indicated that the chronic co-administration of l-NAME (50 or 100 mg/kg/day) with FNZ significantly decreased the time spent by the mice on the rotarod (*p* < 0.001, *p* < 0.0001, respectively) (Fig. 1a) and increased the time spent by the mice in the chimney (*p* < 0.05) (Fig. 1b). Therefore, those results indicate that chronic pretreatment with l-NAME inhibited the development of tolerance to FNZ-induced motor disturbances.

### Influence of Chronic 7-NI Treatment on the Development of Tolerance to FNZ-Induced Motor Disturbances

**Fig. 2:**
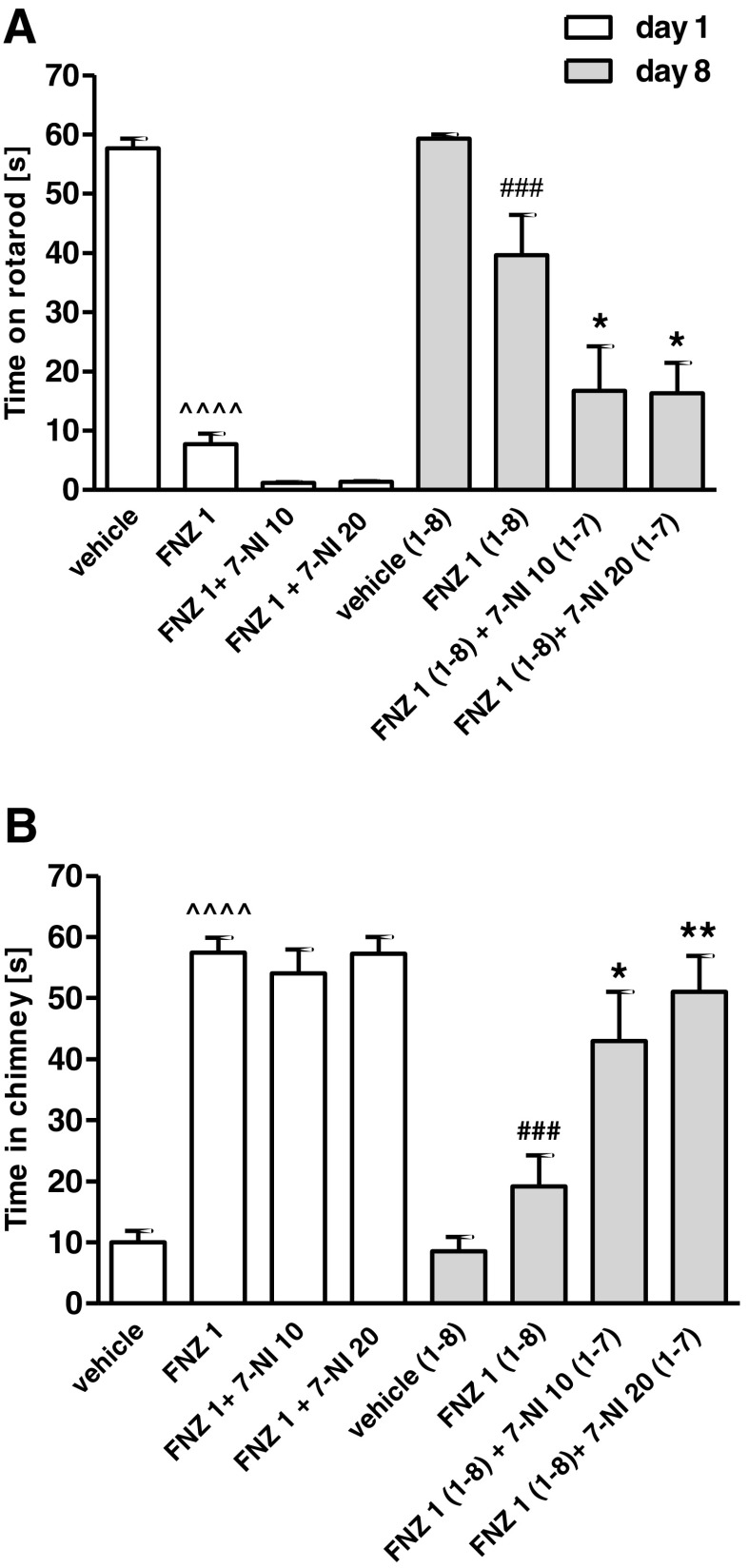
Influence of 7-NI treatment (10 or 20 mg/kg, days 1–7, ip) on the development of tolerance to FNZ-induced motor impairment (1 mg/kg, days 1–8, sc) as assessed by the rotarod test (**a**) and chimney test (**b**). Data represent the mean ± SEM of a group of eight mice (^^^^*p* < 0.0001 vs vehicle; ^###^ *p* < 0.001 vs FNZ 1; **p < 0.01; *p < 0.05 vs FNZ 1 (1–8))

The two-way ANOVA analysis indicated that a repeated administration of FNZ (1 mg/kg/day, 8 days) elicited a significant treatment effect: *F*(3,56) = 64.29, *p* < 0.0001, time effect: *F*(3,56) = 30.07, *p* < 0.0001 and treatment × time interaction: *F*(3,56) = 4.53, *p* = 0065 in the rotarod test (Fig. 2a) and treatment effect: *F*(3,56) = 39.25, *p* < 0.0001, time effect: *F*(3,56) = 20.00, *p* < 000.1 and treatment × time interaction: *F*(3,56) = 6.672, *p* = 0006 in the chimney test (Fig. 2b). The Bonferroni post hoc analysis revealed that the chronic administration of FNZ significantly reduced motor disturbances induced by an acute dose of FNZ, both in the rotarod (*p* < 0.0001) and the chimney tests (*p* < 0.0001). Those results confirm that tolerance was developed to FNZ-induced motor impairment after 8-day treatment.

The Bonferroni post hoc test revealed that the mice on FNZ treatment combined with 7-NI injections (10 or 20 mg/kg) spent significantly less time (*p* < 0.05) on the rotating rod (Fig. 2a) and much time (*p* < 0.05, *p* < 0.01, respectively) in the chimney (Fig. 2b) than the mice receiving only FNZ (Fig. 2b). Therefore, those results reveal that 7-NI inhibited the development of tolerance to the motor-impairing effect of FNZ after chronic co-treatment.

### Influence of Chronic l-Arginine Treatment on the Development of Tolerance to FNZ-Induced Motor Disturbances

**Fig. 3:**
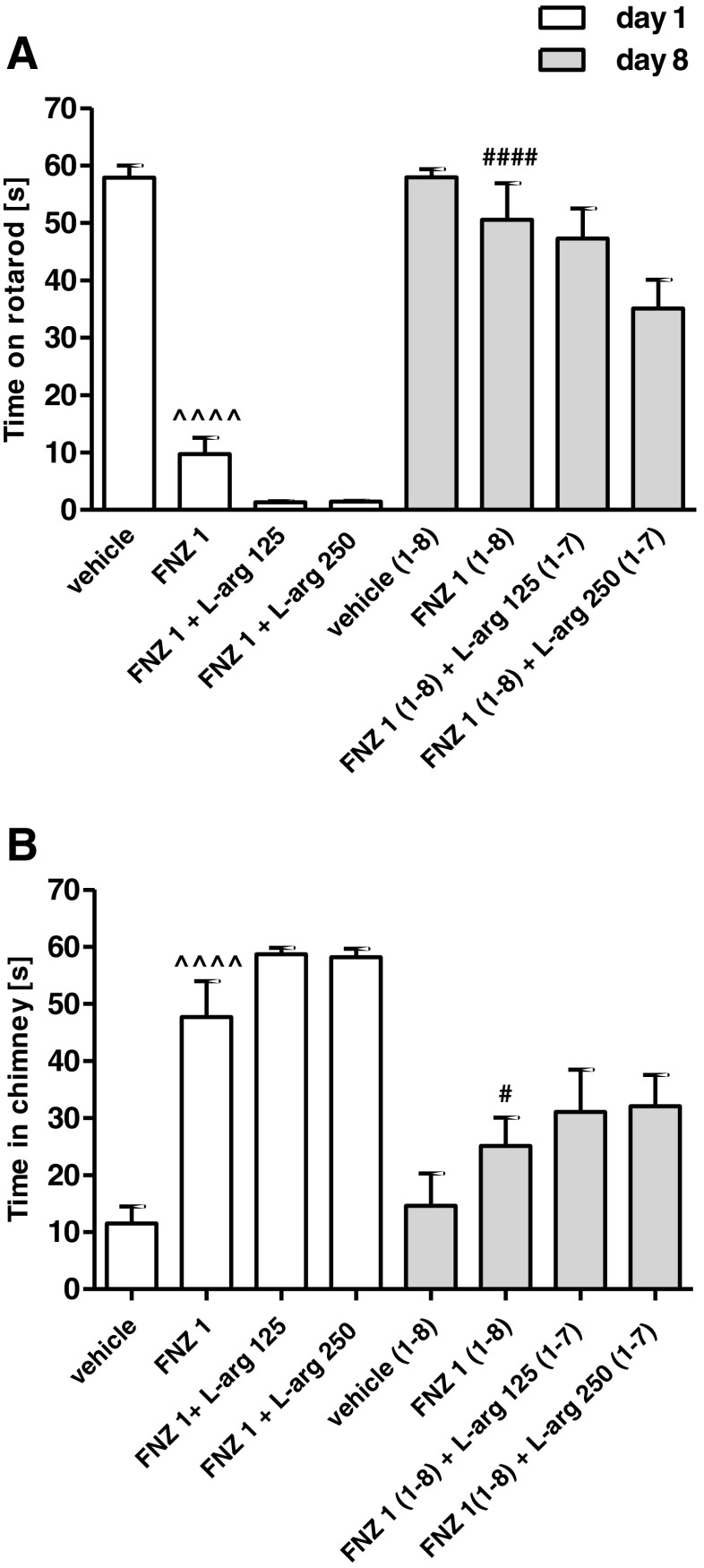
Influence of l-arginine treatment (l-arg; 125 or 250 mg/kg, days 1–7, ip) on the development of tolerance to FNZ-induced motor impairment (1 mg/kg, days 1–8, sc) as assessed by the rotarod test (**a**) and chimney test (**b**). Data represent the mean ± SEM of a group of eight mice (^^^^*p* < 0.0001 vs vehicle; ^####^ *p* < 0.0001; ^#^ *p* < 0.05 vs FNZ 1 mg/kg)

The two-way ANOVA revealed that a repeated administration of FNZ (1 mg/kg/day, 8 days) induced a significant treatment effect: *F*(3,56) = 39.58, *p* < 0.0001, time effect: *F*(3,56) = 69.78, *p* < 0.0001 and treatment × time interaction: *F*(3,56) = 7.923, *p* = 0002 in the rotarod test (Fig. 3a) and treatment effect: *F*(3,56) = 28.24, *p* < 0.0001, time effect: *F*(3,56) = 71.70, *p* < 0.0001 and treatment × time interaction: *F*(3,56) = 8.762, *p* < 0.0001 in the chimney test (Fig. 3b). The Bonferroni post hoc analysis indicated that the chronic administration of FNZ significantly reduced motor disturbances induced by an acute dose of FNZ, both in the rotarod (*p* < 0.0001) and the chimney test (*p* < 0.0001). Those results show that tolerance to the motor-impairing effect was developed after 8-day treatment of FNZ.

As shown in Fig. 3, the Bonferroni post hoc test indicated that the development of tolerance to FNZ-induced motor impairment was not affected by the co-administration of l-arginine (125 and 250 mg/kg), regardless of tested doses. Both rotarod (Fig. 3a) and chimney tests (Fig. 3b) revealed that the 8-day combined therapy resulted in a statistically similar loss of the motor-impairing effects of FNZ to the one observed after the chronic administration of FNZ alone.

### Influence of Chronic Sildenafil Treatment on the Development of Tolerance to FNZ-Induced Motor Disturbances

**Fig. 4:**
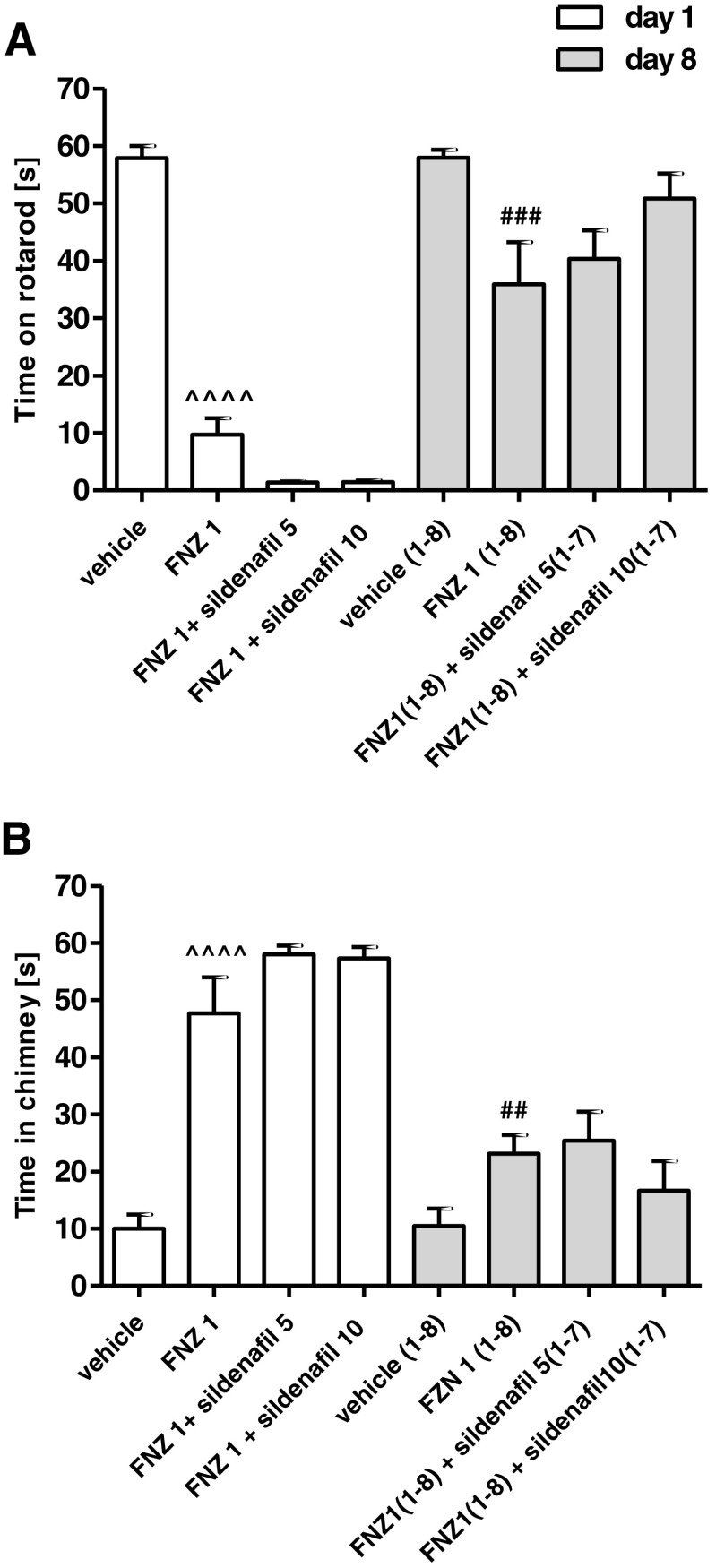
Influence of sildenafil treatment (5 or 10 mg/kg, days 1–7, ip) on the development of tolerance to FNZ-induced motor impairment (1 mg/kg, days 1–8, sc) as assessed by the rotarod test (**a**) and chimney test (**b**). Data represent the mean ± SEM of a group of eight mice (^^^^*p* < 0.0001 vs vehicle; ^##^ *p* < 0.01; ^###^ *p* < 0.001 vs FNZ 1)

The two-way ANOVA analysis indicated that chronic administration of FNZ at a dose of 1 mg/kg/day for 8 days elicited a significant treatment effect: *F*(3,56) = 44.9, *p* < 0.0001, time effect: *F*(3,56) = 115.3, *p* < 0.0001 and treatment × time interaction: *F*(3,56) = 15.12, *p* < 0.0001 in the rotarod test (Fig. 4a) and treatment effect: *F*(3,56) = 36.02, *p* < 0.0001, time effect: *F*(3,56) = 166.8, *p* < 000.1 and treatment × time interaction: *F*(3,56) = 23.20, *p* < 0.0001 in the chimney test (Fig. 4b). The Bonferroni post hoc analysis revealed that the chronic administration of FNZ significantly reduced motor disturbances induced by an acute dose of FNZ, both in the rotarod (*p* < 0.0001) and the chimney tests (*p* < 0.0001). Those results confirm that tolerance was developed to FNZ-induced motor impairment after 8-day treatment.

The Bonferroni post hoc test revealed that co-administration of sildenafil (5 or 10 mg/kg) with FNZ did not change the time spent by the animals on the rotating bar (Fig. 4a) and in the chimney (Fig. 4b). Therefore, both doses of sildenafil had no influence on the development of tolerance to DZ-induced motor disturbances.

### Effect of Acute and Chronic Administration of l-Arginine:NO:cGMP Modulators on Locomotor Activity in Mice

The effect of l-NAME (50 and 100 mg/kg), 7-NI (10 and 20 mg/kg), l-arginine (125 and 250 mg/kg) and sildenafil (5 and 10 mg/kg) on spontaneous locomotor activity in mice is shown in Table 1. Statistical analysis of the results showed that NO modulators used in all tested doses had no statistically significant effect on locomotor activity in mice versus control group, both acutely [one-way ANOVA: *F*(8,71) = 0.7351; *p* = 0.6601] and chronically [one-way ANOVA: *F*(8,71) = 0.8049; *p* = 0.6006] treated.

**Table 1:** Effect of acute and chronic l-arginine:NO:cGMP pathway modulator treatment on locomotor activity. Data represent the mean ± SEM of a group of eight mice (Tukey-Kramer post hoc test)

l-Arginine:NO:cGMP pathway modulators	Locomotor activity/5 min	
	Acute treatment	Chronic treatment
Saline	109.6 ± 6.44	106.5 ± 6.74
l-NAME 50 mg/kg	116.0 ± 14.83	95.8 ± 13.50
l-NAME 100 mg/kg	104.3 ± 14.00	87.0 ± 10.39
7-NI 10 mg/kg	131.6 ± 7.22	91.6 ± 8.24
7-NI 20 mg/kg	112.8 ± 6.15	106.3 ± 8.42
l-Arginine 125 mg/kg	127.4 ± 14.92	109.5 ± 12.23
l-Arginine 250 mg/kg	128.5 ± 9.62	105.8 ± 9.03
Sildenafil 5 mg/kg	119.1 ± 6.85	107.6 ± 7.43
Sildenafil 10 mg/kg	119.6 ± 10.99	92.5 ± 6.62

## Discussion

The major findings of the present study demonstrated that both nonselective NOS inhibitor, l-NAME, and selective nNOS inhibitor, 7-NI, prevented the development of tolerance to the motor impairment effect of FNZ, both in the rotarod and chimney tests in mice. It is worth emphasizing that the doses of drugs modifying l-arginine:NO:cGMP pathway used in the present experiments were chosen on the base of our previous experiments and both acute and chronic administration of NO modulators did not affect the motor performance in mice (Talarek et al. 2008; Talarek et al. 2010). Therefore, the results of the present study seem not to be associated with motor-impairing effect of these compounds per se. Furthermore, in order to avoid the risk of obtaining the false effects caused by a possible influence of the NO modulators on the locomotor activity, motility of mice was measured in our study. The results showed that both acute and chronic administration of NO modulators used in all tested doses had no statistically significant effect on locomotor activity in mice.

The present studies also showed that NO precursor, l-arginine, and a potent PDE5 inhibitor, sildenafil, did not affect the development of tolerance to the motor impairment effect of FNZ in both behavioural tests. The lack of effect of l-arginine and sildenafil on the tolerance to FNZ is difficult to explain. Some studies have shown that l-arginine up to 1000 mg/kg was effective without impairing open-field locomotor activity in mice (Ulusu et al. 2005; Uzbay et al. 2007). Therefore, it is possible that too low, inefficient doses of the NO precursor (up to 250 mg/kg) would account for the lack of effect of l-arginine on the tolerance to FNZ in the present experiments.

Till now, there is little data on the involvement of l-arginine:NO:cGMP system in BZ tolerance. Our previous studies with DZ, classical BZ, commonly used in clinical practice, showed the inhibition of DZ-induced tolerance to the motor-impairing effect after chronic pretreatment with l-NAME and 7-NI (Talarek et al. 2008). Interestingly, we also indicated the facilitation of DZ-induced tolerance to the motor-impairing effect in the rotarod test after chronic pretreatment with l-arginine and sildenafil (Talarek et al. 2008; Talarek et al. 2010). In contrast, Nidhi et al. (2000) reported that administration of nonselective NOS inhibitor—*N* ^G^-nitro-l-arginine (l-NOARG)—did not prevent the development of tolerance to the anticonvulsant effects of DZ in rats. What is more, they demonstrated that administration of l-arginine combined with DZ inhibited tolerance developed after prolonged use of DZ. The discrepancies between these studies could be due to the various mechanisms involved in the development of tolerance to different effects of BZ. Tolerance to BZ diverse effects (i.e. sedative effects, motor disturbances or anxiolytic effects) after a prolonged treatment is a well-documented issue, demonstrated in many animal studies (Ferreri et al. 2015; Talarek et al. 2008; Talarek et al. 2010; Vinkers and Olivier 2012). However, this diminution in the response to the drug does not occur simultaneously for every pharmacological property. Tolerance to the sedative effects of BZ occurs more rapidly than to anticonvulsant effects, with the anxiolytic effects in animals at least, occurring after an even longer time period (Bateson 2002; Ferreri et al. 2015; Gravielle 2016; Vinkers and Olivier 2012). These observations suggest that different mechanisms are responsible for the development of tolerance to each of these drug-induced effects and/or different brain regions and BZ receptor subunits are involved in these mechanisms (Bateson 2002; Ferreri et al. 2015; Gravielle 2016). Furthermore, emergence of tolerance to BZ appears to be dependent on the route of drug administration, animal test studied and treatment schedule. It has been suggested that different treatment regimens during chronic BZ administration produced significantly different adaptive changes at specific GABA_A_ receptors (Fernandes et al. 1999). Moreover, the discrepancies between studies that investigated the phenomenon of tolerance may be due to differences in pharmacological properties of different BZ (Hauser et al. 1997; Sankar 2012). Hauser et al. (1997) reported that FNZ can act as either an agonist or an inverse agonist, depending on GABA_A_ configuration. It is also known that FNZ produces relatively strong sedation and amnesia in humans, in comparison with other compounds from the same pharmacological group. These properties have promoted its abuse as a date-rape drug. It should be noted that FNZ is 10 times as potent as DZ (Ramadan et al. 2013). Interestingly, in our previous study, we showed that an acute dose of FNZ (1 mg/kg, *sc*) enhanced locomotor activity of mice, whereas the injection of DZ in mice on the first day of that experiment induced sedation (Talarek et al. 2013). Other classic BZ—clonazepam and chlordiazepoxide—also provoked sleep after acute administration in mice (Talarek and Fidecka 2004). Moreover, behavioural studies of Orzelska et al. (2013, 2015) with a comparison of DZ and FNZ indicated that NOS inhibitors differentially affected DZ and FNZ responses of rats in the novel object recognition test. These results seem to be pertinent because it can be supposed that different BZ compounds will differ in terms of their side effects and in terms of interactions with drugs that modify the level of NO.

The mechanisms by which the modification of the l-arginine:NO:cGMP pathway affects BZ tolerance are not clear. It is suggested that chronic treatment with NOS inhibitors and FNZ would lead to prevention of GABA_A_ receptor downregulation and/or glutamate receptor upregulation. There is evidence that NO is produced following activation of *N*-methyl-d-aspartate (NMDA) receptors (Garthwaite 2008; Uzbay and Oglesby 2001). Interestingly, many studies reveal the ability of NMDA receptor antagonists to attenuate tolerance to motor-impairing effects after long-term treatment with various drugs acting via the GABA_A_ receptors. Our previous study showed that memantine and ketamine, the non-competitive antagonists of the NMDA receptor complex, decreased the development and/or expression of DZ tolerance in mice (Talarek et al. 2016). Therefore, it is possible that NO interactions with the glutamatergic neurotransmission may play role in the effects of NOS inhibitors on FNZ-induced tolerance, observed in our present experiments. However, further studies are required to clarify the precise mechanisms underlying our findings, because the presence of other interactions in the CNS could not be excluded.

In conclusion, our data indicate that the non-selective NOS inhibitor, l-NAME, and the selective nNOS inhibitor, 7-NI, prevented the development of tolerance to the motor impairment effect of FNZ, both in the rotarod and chimney tests in mice. The present study also demonstrates that a NO precursor, l-arginine, and a potent PDE5 inhibitor, sildenafil, did not affect the development of tolerance to the motor impairment effect of FNZ. The results of our investigation have provided behavioural evidence that l-arginine:NO:cGMP pathway may contribute an important role in the development of FNZ tolerance. This conclusion has an important clinical implication because FNZ abuse is very common and understanding the interaction of FNZ with the drugs modifying the level of NO, such as nitrovasodilators or drugs used for erectile dysfunction, might be very useful for the psychiatric assessment.
